# Numerical Simulations of the Superfluid $^3$He Order Parameter Using dyGiLa: A Homogeneous Rapid Quench to B Phase

**DOI:** 10.1007/s10909-026-03428-1

**Published:** 2026-06-15

**Authors:** Asier Lopez-Eiguren, Mark Hindmarsh, Kari Rummukainen, Kuang Zhang

**Affiliations:** 1 Department of Physics, University of the Basque Country UPV/EHU, 48080 Bilbao, Spain; 2 EHU Quantum Center, University of the Basque Country UPV/EHU, Leioa, 48940 Biscay Spain; 3 Department of Physics and Astronomy, University of Sussex, Falmer, Brighton, BN1 9QH UK; 4 Department of Physics and Helsinki Institute of Physics, University of Helsinki, PL 64, 00014 Helsinki, Finland

**Keywords:** Helium 3, Phase transitions, Time-dependent Ginzburg–Landau equation, Cosmology, Early universe, Gravitational waves

## Abstract

The first-order A–B phase transition in superfluid $^3$He remains poorly understood, as homogeneous nucleation theory predicts negligible transition rates, while experiments observe rapid and reproducible phase conversion. Recent studies, including confined geometries and controlled supercooling experiments, point to the importance of non-equilibrium dynamics and localized energy deposition. In this work, we present dyGiLa, a massively parallel numerical framework for simulating the real-time evolution of the superfluid order parameter using time-dependent Ginzburg–Landau (TDGL) theory. The code evolves both the order parameter and the temperature field self-consistently, enabling the study of quenches and other non-equilibrium processes on large three-dimensional lattices. We demonstrate the capabilities of the framework through large-scale quench simulations, where we observe the dynamical emergence of the B phase, along with B–B domain walls and vortices. The onset of B-phase formation is consistent with a Kibble–Zurek-type mechanism, providing a natural pathway for phase conversion beyond homogeneous nucleation. These results represent a step toward a dynamical understanding of the A–B transition and establish superfluid $^3$He as a laboratory for studying non-equilibrium phenomena relevant to cosmological phase transitions.

## Introduction

Phase transitions are important in many areas of physics, from the early Universe to the low-temperature behavior of quantum fluids. In cosmology, they can contribute to baryon number generation [1–3] and are sources of stochastic gravitational wave backgrounds, with a characteristic frequency set by the critical temperature. Such signals are targets of current and future gravitational wave detectors, and their detection would provide a direct probe of the early Universe. In first-order phase transitions, the transition proceeds via the nucleation of bubbles of the stable phase after supercooling. In the standard approach, the transition rate is computed using homogeneous nucleation theory [4, 5], adapted to relativistic quantum field theory [6, 7]. The properties of the resulting gravitational-wave signals can be described using a small set of equilibrium and near-equilibrium quantities, among which the transition rate plays a central role [8].

In order to distinguish the cosmological background from astrophysical foregrounds, the signal must be accurately characterised, and the production process well understood. Laboratory analogues have already shown themselves to be of great importance in understanding the dynamics of phase transitions, as demonstrated by the Kibble-Zurek mechanism [9–11] for topological defect formation. In cosmology, symmetry-breaking phase transitions that take place as the Universe cools down can give rise to a variety of defects, such as domain walls, cosmic strings or monopoles. The same mechanism operates in condensed matter systems, and accounts for the vortex production observed in rapid quenches, quenches with timescale shorter than the characteristic relaxation time of the order parameter, in superfluid $^3$He [12–18]. This provides strong support for the idea that if topological defects exist in a theory, they will form at a phase transition in the early universe, with important observational consequences, including gravitational waves.

Superfluid $^3$He also has a first-order phase transition between two superfluid phases, A and B. According to homogeneous nucleation theory, the nucleation rate is exponentially suppressed by the free-energy cost associated with forming an interface between the two phases, leading to extremely long-lived supercooled states when the free-energy barrier is large. The standard theory therefore predicts exceedingly long lifetimes for the metastable A phase below the equilibrium A–B transition temperature.

Despite the success of homogeneous nucleation theory in idealized settings, the experimentally observed A–B phase transition in superfluid $^3$He remains poorly understood. Theoretical estimates from homogeneous nucleation theory predict nucleation rates that are negligibly small on experimental timescales, while laboratory experiments consistently observe the transition to occur rapidly and reproducibly under a wide range of conditions [19–28]. This discrepancy suggests the presence of additional mechanisms that enhance the transition probability. A variety of explanations have been proposed [25, 28–35], including triggering by ionizing radiation, surface- and boundary-induced nucleation, and the involvement of textural singularities or other nontrivial configurations of the order parameter. While each of these mechanisms may play a role under specific circumstances, none has yet provided a comprehensive and universally accepted explanation for the observed robustness of the transition, highlighting the need for a fully dynamical, non-equilibrium description of the phase transition process.

Due to the close correspondence between the symmetry-breaking patterns and order-parameter structures in cosmology and in superfluid $^3$He, this system provides a unique analogue platform for studying non-equilibrium dynamics, defect formation, and phase transition kinetics that are otherwise inaccessible in the early Universe. In particular, the first-order A–B transition shares key features with cosmological first-order transitions, including metastability, supercooling, and the presence of an energy barrier separating phases. Therefore, the discrepancy observed between theory and experiment in A–B transition is of particular significance for cosmology. More so if it is taken into account that all current predictions of gravitational-wave spectra from first-order phase transitions rely on relativistic generalizations of classical nucleation theory.

These deep structural and dynamical analogies motivate the application of numerical techniques originally developed for cosmological phase transitions to the study of superfluid $^3$He. By exploiting this cross-disciplinary approach, superfluid $^3$He can be used not only to address its own long-standing nucleation puzzle, but also to inform and refine theoretical predictions for phase transitions and gravitational-wave production in the early Universe.

In this work, we adopt a cosmology-inspired computational technique to investigate phase transition dynamics in superfluid $^3$He, following the first steps described in [20]. By performing large-scale numerical simulations based on a time-dependent Ginzburg–Landau (TDGL) description [36–38], we aim to understand non-equilibrium phenomena such as domain formation, phase ordering, and the emergence and evolution of topological defects, which may play a central role in driving the transition beyond the predictions of homogeneous nucleation theory. Our approach uses numerical techniques originally developed for cosmological phase transitions, where similar dynamics have already been analyzed across wide ranges of length and time scales.

In this sense, the present manuscript focuses on the implementation and validation of the numerical framework, while also highlighting its broader potential. Accordingly, we present the code and describe several of its capabilities. At the same time, we demonstrate that key physical mechanisms are already captured within this approach, including signatures consistent with the Kibble–Zurek mechanism, such as the non-equilibrium emergence of the B-phase, as well as the formation of B–B domain walls and vortices [39–41].

This work is carried out within the framework of the QUEST-DMC (Quantum-Enhanced Superfluid Technologies for Dark Matter and Cosmology) collaboration, established under the UK Quantum Technology for Fundamental Physics programme. One of the objectives of QUEST-DMC is to address the long-standing nucleation problem in superfluid $^3$He by combining state-of-the-art experimental capabilities with advanced theoretical and computational tools that were not available at the time of the pioneering experiments of the 1990s. By integrating modern high-performance computing, refined theoretical modeling, and insights drawn from cosmology, the collaboration seeks to develop a unified and quantitative understanding of phase transition dynamics in superfluid $^3$He and its broader implications for fundamental physics.

## Theoretical Framework

Our theoretical framework for describing the dynamics of superfluid $^3$He is time-dependent Ginzburg–Landau (TDGL) theory. In the following subsection we will summarize the theory as applied to $^3$He. For more detailed description we refer the reader to [20].

### Time-Dependent Ginzburg–Landau Model for $^3$He

The TDGL approach treats the superfluid order parameter as a bosonic field whose dynamics capture the collective behavior of Cooper pairs, while incorporating dissipation and fluctuations arising from the underlying fermionic degrees of freedom. This theory provides a controlled and symmetry-respecting description of phase ordering, defect formation, and nucleation phenomena near the critical temperature and into the metastable regime.

The order parameter of superfluid $^3$He is a complex 3$\times$3 matrix, $A_{\alpha i}({\textbf {r}},t)$, reflecting spin-triplet ($S=1$) and orbital-triplet ($L=1$) pairing. It transforms as a vector under independent spin and orbital rotations and carries a global U(1) phase associated with particle number conservation. The full symmetry group of the normal phase includes continuous rotations in spin and orbital space, global gauge transformations, and discrete symmetries such as time reversal, parity, and particle–hole conjugation. The TDGL formulation preserves these symmetries at the level of the effective action, which is derived by integrating out the underlying fermionic degrees of freedom (see e.g. [42, 43]).

The static part of the free energy density has the familiar Ginzburg–Landau form

1 $$\begin{aligned} f_\text {GL}= &   \alpha \,\text{ Tr }\left( A\,A^{\dagger }\right) - \sum _{p=1}^{5}\beta _{p\,}u_{p}(A) - \sum _{m=1}^{3}\,K_{m}\,v_{m}(\partial A) , \end{aligned}$$

where the quadratic term controls the instability toward superfluidity, the quartic interaction terms $u_p$ select the equilibrium phase, and the gradient terms $v_m$ penalize spatial variations of the order parameter. The effect of spin-orbit coupling induces an extra quadratic terms. This dipole pairing contribution can be neglected on length scales less than $7-8\,\mu$m.

The parameters $\alpha$, $\beta _p$ and $K_m$ are temperature- and pressure-dependent and they can be obtained from the microscopic theory of superfluid $^3$He [36, 44–47]. Near the critical temperature, they are

2 $$\begin{aligned} \alpha (T) = \frac{1}{3}N(0)(T/T_\text {c}- 1), \end{aligned}$$

and

3 $$\begin{aligned} \beta _p = \beta _0\left( b^\text {wc}_p + \frac{T}{T_\text {c}} b^\text {sc}_p\right) \,,\quad p\in \{1,\ldots , 5\} \end{aligned}$$

where

4 $$\begin{aligned} \beta _0 = \frac{7\zeta (3)}{80\pi ^2} \frac{N(0)}{3 (k_\text {B}T_\text {c})^2} \,. \end{aligned}$$

and *N*(0) is the density of states per spin at the Fermi surface.

The five quartic couplings $\beta _p$ include both weak-coupling contributions, $b^\text {wc}_p$, and strong-coupling corrections, $b^\text {sc}_p$, arising from quasiparticle interactions in the normal Fermi liquid. The weak coupling limit take the values, $\{b^\text {wc}_p\} = (-1, 2, 2, 2, -2)$. The strong-coupling effects are computed based on the leading order corrections to weak-coupling BCS theory as formulated by Rainer and Serene [44] and computed most accurately by Wiman and Sauls [48], see also [49, 50]. This parameters can be also extracted from experiments [51]. They are essential for correctly reproducing the stability of the A phase at high pressures and for determining the equilibrium A–B transition line.

Spatial gradient terms, controlled by $K_m$ parameters, play a central role in the dynamics of interfaces and defects. These parameters, can be calculated to good approximation in weak-coupling theory,

5 $$\begin{aligned} K_1 = K_2 = K_3 =\frac{7\zeta (3)}{60} N(0) \xi _{0}^{2} \,, \end{aligned}$$

where $\xi _{0}=\hbar v_{\text {F}}/2 \pi k_\textrm{B} T_c$ is the zero-temperature Cooper pair correlation length. They determine the stiffness of the order parameter against distortions and set the characteristic energy scale of textures, vortices, and domain walls. These terms are crucial for describing the structure and energetics of the A–B interface, which in turn governs the nucleation barrier in first-order transitions.

The effect of a external magnetic field, $\textbf{H}$, which changes the equilibrium phase diagram of superfluid $^3$He [50], can be accounted adding two magnetic field coupling terms to the GL free energy (1). These terms are

6 $$\begin{aligned} f_H=g_H H_{\alpha } (AA^{\dagger })_{\alpha \beta }H_{\beta } + g_z i \epsilon _{\gamma \alpha \beta }(AA^{\dagger })_{\alpha \beta }H_{\gamma }. \end{aligned}$$

The most important term, except very close to $T_\text {c}$, is the term quadratic in the field, whose coupling constant is

7 $$\begin{aligned} g_H=20 \beta _0 \left( \frac{\mu _h}{1+F_0^a}\right) ^2, \end{aligned}$$

where $\mu _h\simeq -1.07 \times 10^{-26}$ J T$^{-1}$ is the helion magnetic moment and $F_0^a \simeq -0.7$ is the exchange interaction parameter [38].

The TDGL equation is obtained from the effective action in an expansion in time derivatives and takes the form of coupled nonlinear wave equations for the components of $A_{\alpha i}({\textbf {r}},t)$. One can account for thermal fluctuations of the fermionic quasiparticle bath with a stochastic Langevin noise source, in which case the equation takes the form

8 $$\begin{aligned} \begin{array}{rcl} \tau _0\ddot{A}_{\alpha i} & +&  \gamma \dot{A}_{\alpha i} + \alpha A_{\alpha i} - K_1\partial ^2 A_{\alpha i} - (K_2+K_3) \partial _i\partial _j A_{\alpha j} \\ & &  + g_H H_\alpha H_\beta A_{\beta i}+ i g_z H_{\gamma }\epsilon _{\gamma \alpha \beta }A_{\beta i} \\ & &  + 2\left[ \beta _1 A^*_{\alpha i} \text{ Tr }\left( A A^T\right) + \beta _2 A_{\alpha i} \text{ Tr }\left( A A^\dagger \right) \right. \\ & &  + \quad \left. \beta _3 (A A^T A^*)_{\alpha i} + \beta _4 (A A^\dagger A)_{\alpha i} + \beta _5 (A^* A^T A)_{\alpha i} \right] = \zeta _{\alpha i}({\textbf {r}},t) \,. \end{array} \end{aligned}$$

where $\tau _0$ is an effective inertia parameter, $\gamma$ is a damping term and $\zeta _{\alpha i}({\textbf {r}},t)$ is a stochastic thermal noise source. The inertia parameter is fixed by the frequency of Higgs modes in the B phase to $\tau _0 = 5\hbar ^2\beta _0$ [52], while the damping parameter takes the value $\gamma = \hbar \pi N(0)/48k_\text {B}T_\text {c}$ at the critical temperature, and goes to zero as temperature decreases with the density of quasiparticles. While the density of quasiparticles is different in A and B phase, a good approximation to both for $T/T_\text {c}\gtrsim 0.5$ is $(T/T_\text {c})^4$, which we use. The noise fluctuation amplitude and the damping are related by the fluctuation-dissipation theorem in the usual way. Including the noise enables the study of thermally activated processes and non-equilibrium relaxation.

Close to the critical temperature, dissipation dominates the dynamics and the system exhibits overdamped behavior, while at lower temperatures inertial effects become increasingly important. Retaining both contributions allows the TDGL framework to interpolate between diffusive and propagating regimes of order-parameter dynamics. This is particularly relevant for the study of rapid quenches, localized energy deposition, and defect-mediated nucleation scenarios.

Overall, the TDGL formulation provides a powerful and flexible theoretical framework for simulating the space–time evolution of the superfluid $^3$He order parameter under non-equilibrium conditions. By capturing the correct symmetry structure, incorporating strong-coupling effects, and allowing for dissipation and fluctuations, it enables quantitative investigations of nucleation pathways, defect formation, and phase transition dynamics. These features make TDGL theory ideally suited for addressing the long-standing A–B nucleation puzzle and for drawing direct analogies with first-order phase transitions in the early Universe.

## Numerical Implementation

The numerical simulations presented in this work are performed within a lattice-based real-time evolution framework. The underlying numerical infrastructure is based on the dyGiLa code [53], whose earlier version was already used in the first simulations reported in [20]. Building on this framework, the new elements introduced in this work concern the construction of the initial conditions and the implementation of the boundary conditions, which are formulated to allow for a wide range of distinct configurations, enabling the simulation of different experimental and laboratory setups. In this section, we provide an overview of the numerical approach, with the details of the code, initial conditions, and boundary conditions discussed separately in the following subsections.

### The dyGiLa Code

Simulating an order parameter with 3$\times$3 complex components in 3+1 dimensions requires a huge amount of computational resources, both in terms of parallel floating-point performance and efficient input/output (I/O) capabilities for data analysis and visualization. Over the past two decades, advances in high-performance computing (HPC) have significantly improved both hardware architectures and supporting software ecosystems, enabling large-scale real-time simulations of complex field dynamics.

The numerical framework employed in this work builds on these developments and follows a formulation similar to that used in earlier studies. The time-dependent Ginzburg–Landau equations shown in Eq. (8) are discretized using finite-difference schemes in space and explicit time integration [54], and are evolved on a three-dimensional Cartesian lattice. The equations are expressed in dimensionless form. In order to do so, the order parameter $A_{\alpha i}$ is expressed in units of $k_\textrm{B} T_c$, the length unit is the zero-temperature limit of the GL coherence length $\xi _\text {GL}=\sqrt{\zeta (3)/10}\xi _0$, and the time unit is $t_\text {GL}^B=\sqrt{5/3}(\xi _\text {GL}/v_F)$, where $v_F$ is the Fermi velocity. The bulk free energy term linear in the magnetic field and the dipole pairing terms are not yet implemented, as we investigate length scales of around O(1) $\mu$m sufficiently far from $T_\text {c}$.

Since the coefficients of the TDGL equations depend explicitly on the local temperature, dyGiLa treats the temperature as a dynamical field that is evolved self-consistently alongside the order parameter. This feature allows the simulation of both static thermal backgrounds and time-dependent temperature evolutions, such as homogeneous quenches, controlled cooling protocols, or localized heating events. As a result, the code can naturally capture the coupled dynamics of temperature and order-parameter fields during phase transitions.

To efficiently utilize distributed-memory HPC systems, the simulations are implemented within the HILA lattice field theory framework [55, 56], which provides a uniform Cartesian grid with flexible resolution along each spatial direction. HILA offers a set of pre-defined data structures for handling scalar, vector, and tensor fields on the lattice and supports efficient domain decomposition and parallel execution. This infrastructure underlies the dyGiLa code and enables simulations on large three-dimensional grids while maintaining good scaling with system size and processor count. HILA also provides the capability to run on a wide range of supercomputing architectures, including GPGPU-based systems.

### Initial Conditions

In the following, we discuss the initial conditions employed in the simulations. Since dyGiLa evolves both the order parameter and the temperature field dynamically, initial conditions must be specified consistently for both quantities. We therefore describe below the choices adopted for initializing the order-parameter field and the temperature distribution, which together define the physical state of the system at the start of the evolution.

#### Order Parameter

The order-parameter field can be initialized in a variety of physically motivated configurations, allowing for a controlled exploration of different phase-transition scenarios. Uniform initial conditions corresponding to the A-phase, B-phase, or the normal phase can be imposed throughout the simulation volume. In addition, small-amplitude random noise can be superimposed on these homogeneous configurations in order to seed instabilities and mimic thermal or experimental fluctuations present at the onset of the evolution.

**Fig. 1:**
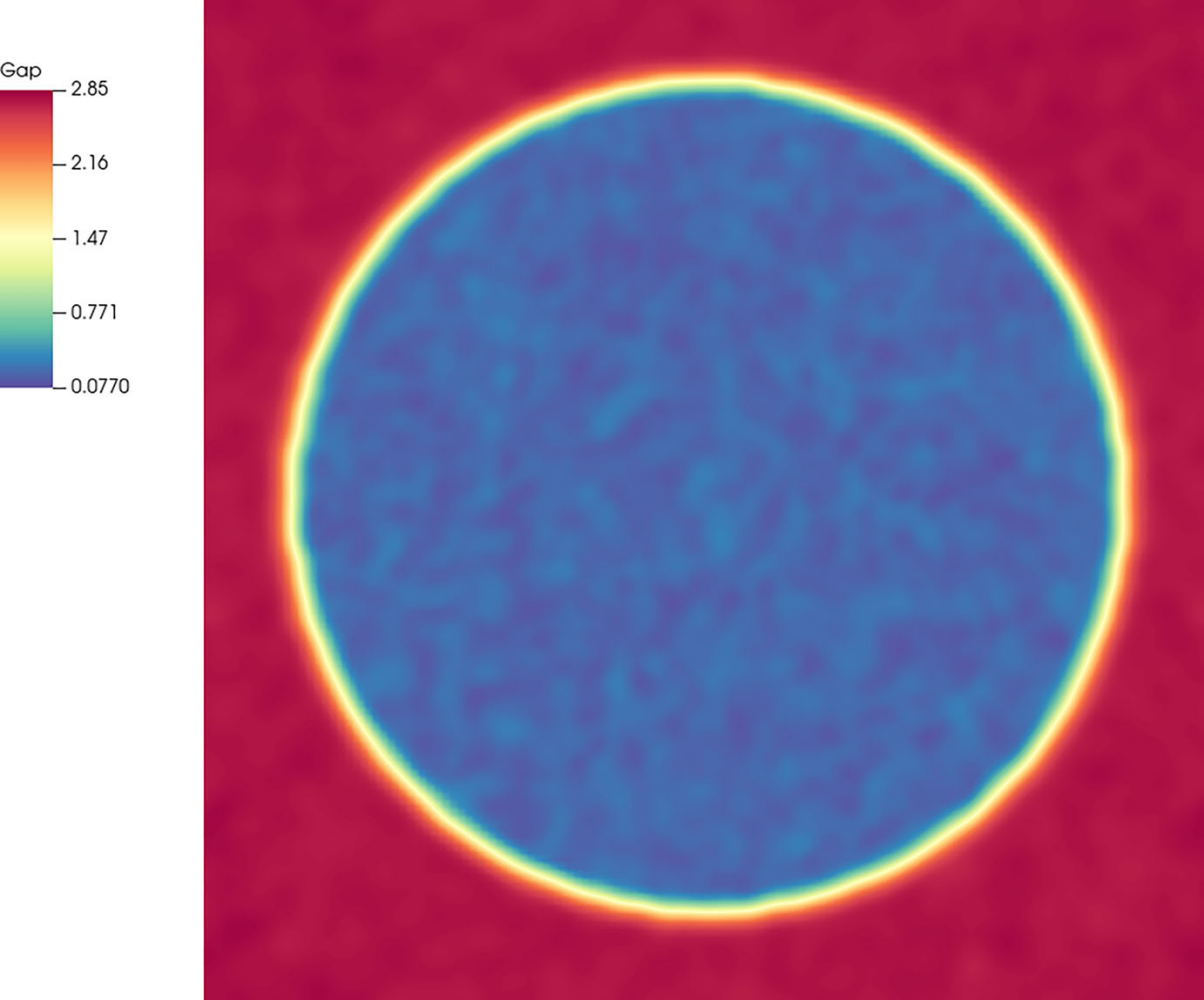
Initial condition for the order parameter. The system is initialized with a normal (gapless) phase at the center, surrounded by the A phase. The color scale represents the magnitude of the superfluid energy gap: blue corresponds to zero gap (normal phase), while red indicates the maximum gap characteristic of the A phase. The figure shows a central slice through the simulation domain along one spatial direction, passing through the center of the normal region

Inhomogeneous initial conditions are also supported, where the simulation volume is partitioned into regions initialized in different phases. A commonly used configuration consists of a spherical region initialized either in the B-phase or in the normal phase, embedded within an A-phase background. The radius, position, and phase assignment of the embedded region can be freely specified. Such configurations are particularly useful for studying bubble growth, phase conversion, and interface dynamics between competing superfluid phases. For example, in Fig. 1 an spherical normal phase bubble initialised in the center of a A-phase region is shown.

#### Temperature

Since the temperature field is evolved dynamically in dyGiLa, initial conditions must also be specified for the temperature. The simplest choice corresponds to a uniform temperature profile, which can be set either above or below the critical temperature depending on the physical scenario under consideration. This setup is typically used to study homogeneous quenches or relaxation processes in the absence of spatial temperature gradients.

**Fig. 2:**
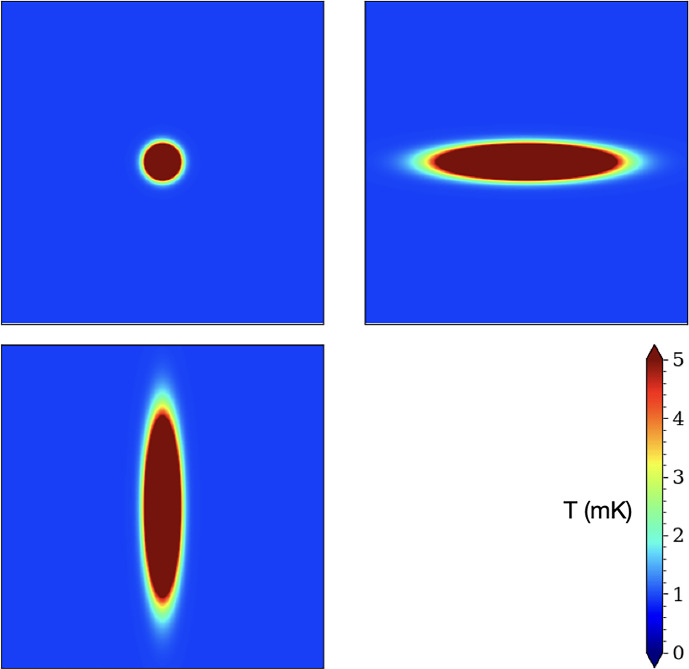
Initial condition for the temperature field. The system is initialized with a cigar-shaped hot region embedded in a colder background. The color scale indicates temperatures ranging from 0 to 5 mK, with blue corresponding to 0 mK and red to the maximum temperature of 5 mK. Shown are central slices through the simulation domain in the three spatial directions, all passing through the center of the hot region

To model localized heating and non-uniform thermal environments, spatially varying temperature profiles can also be initialized. In particular, the temperature field can be initialized using a three-dimensional Gaussian profile, allowing for the creation of localized hot regions. By choosing isotropic Gaussian widths, a hot spherical region is obtained, while anisotropic widths lead to elongated structures, referred to as hot cigar configurations, as shown in Fig. 2. The amplitude, size, orientation, and position of these thermal structures can be independently controlled. These temperature initial conditions provide a flexible way to mimic experimentally relevant situations such as localized energy deposition, focused heating, or non-equilibrium cooling protocols, and to study their impact on the coupled evolution of temperature and order-parameter fields.

### Boundary Conditions

We next discuss the boundary conditions employed in the simulations. Since dyGiLa evolves both the order parameter and the temperature field dynamically, boundary conditions must be specified for each of these quantities. These conditions play an important role in determining the evolution of the system, particularly in confined geometries or in situations where thermal gradients or phase-selective surfaces are present.

A key feature of the numerical implementation is that boundary conditions can be chosen independently on each face of the cubic simulation domain, both for the order parameter and for the temperature. This flexibility allows for the construction of asymmetric or mixed boundary configurations, enabling the modeling of a wide range of experimental environments. In the following, we describe the different boundary-condition options available for each field.

#### Order Parameter

Several physically motivated boundary conditions are available for the order-parameter field. Periodic boundary conditions allow for the study of bulk-like systems without explicit boundaries. In contrast, fixed-phase boundary conditions can be imposed, where the order parameter at the boundary is constrained to correspond to the A-phase, B-phase, or the normal phase. These conditions are useful for modeling phase-selective surfaces or interfaces with external reservoirs.

To represent strongly pair-breaking boundaries, a maximal pair-breaking condition is also implemented, in which the order parameter is suppressed at the boundary ($A_{\alpha i}=0$). In addition, Robin-type boundary conditions are supported, controlled by a tunable parameter $b_T$, which interpolates between Neumann- and Dirichlet-like behavior. For a boundary normal to the z-direction, these conditions take the form

9 $$\begin{aligned} A_{\alpha z}|_{z=0}=0, \qquad \nabla A_{\alpha x}|_{z=0}=\frac{1}{b_T}A_{\alpha x}|_{z=0}, \qquad \nabla A_{\alpha y}|_{z=0}=\frac{1}{b_T}A_{\alpha y}|_{z=0}. \end{aligned}$$

By varying $b_T$, the strength of pair breaking at the boundary can be continuously tuned. As with the temperature field, different order-parameter boundary conditions can be applied independently on each face of the simulation domain, allowing for the construction of complex boundary environments relevant to confined superfluid $^3$He experiments.

#### Temperature

For the temperature field, two types of boundary conditions are implemented. Periodic boundary conditions can be imposed to model effectively infinite or translationally invariant systems, minimizing boundary effects. Alternatively, fixed-temperature (Dirichlet) boundary conditions can be applied, where the temperature at a given face of the simulation volume is held at a prescribed value. This option allows for the study of systems subject to external thermal control, such as cooling through container walls or imposed temperature gradients across the sample.

Each face of the simulation volume can be assigned its own temperature boundary condition, enabling configurations where, for example, heat is injected or extracted through selected boundaries while others remain periodic. This provides a flexible way to model non-equilibrium thermal environments relevant to laboratory experiments.

## Results

We begin by presenting results from simulations of a homogeneous rapid quench, which serve as a baseline for validating the numerical framework and for isolating purely bulk, non-equilibrium effects in the absence of spatial temperature gradients or boundary-induced asymmetries. In these simulations, the system is initialized at a uniform temperature above the superfluid transition and subsequently quenched homogeneously to a final temperature below $T_\text {c}$ over a time $\tau _q$. A rapid homogeneous quench models the interior of a small region of the superfluid which has been heated above $T_\text {c}$ by an energetic particle such as a neutron [16, 17, 33]. Understanding the subsequent dynamics is important for the study of topological defect formation and assessing solutions to the A- B nucleation puzzle invoking such interactions [29, 33, 57].

The simulation was performed in a spatial grid with 512$\times$512$\times$512 sites with lattice spacing $\xi _{GL}$, we have checked that variations in volume and lattice spacing change the average value of the gap ($\Delta = \sqrt{\text {tr} A^\dagger A}$) by less than 5 $\%$. The pressure was set to $p=5.5$ bar, which was chosen to be relevant for A–B transition experiments in the set-up described in Ref. [19], in which most data is taken at this pressure. The results will be reported elsewhere [58].

The pressure 5.5 bar is below the tricritical point and one expects to see a direct transition between the normal phase and the B phase, for which $\xi _{GL}=26.30$ nm and $t_\text {GL}^B=0.52$ ns, making the length of the side of our cube 13.47 $\mu$m. The simulation was evolved 1000$t_\text {GL}^B$, which corresponds to 520 ns. The damping parameter was set to $\gamma =2.95$ in dimensionless units. For this simulation, we choose $\textbf{H}=(0.3,0,0)$ T.

The simulation starts with a homogeneous temperature set at $T_\textrm{i}=7$ mK and the order parameter is initialized in the normal phase. After a short period of 5.2 ns at 7 mK the system starts to cool down homogeneously with a constant rate. The quench timescale used is $\tau _q=50\ T_\text {c}$, which is shorter than the Ginzburg–Landau relaxation time for this system. Therefore, the quench can be considered rapid. This cooling is performed for 101.8 ns or until the system reaches $T_\textrm{f}=0.7\ T_\text {c}$. For this pressure $T_\text {c}=1.52$ mK. Afterwards, the system is evolved with constant temperature until the end. The temperature evolution is described by the following equation:

10 $$\begin{aligned} T(t)= {\left\{ \begin{array}{ll} T_\textrm{i} &  \text {if } t< 5.2 \text { ns} \\ T_\textrm{i}-\frac{T_\text {c}}{\tau _q}t &  \text {if } 5.2 \text { ns} \le t < 101.8 \text { ns} \\ T_\textrm{f} &  \text {if } t \ge 101.8 \text { ns} \end{array}\right. } \end{aligned}$$

Throughout this process, periodic boundary conditions are imposed on both the order parameter and the temperature field across all six faces of the simulation domain.

**Fig. 3:**
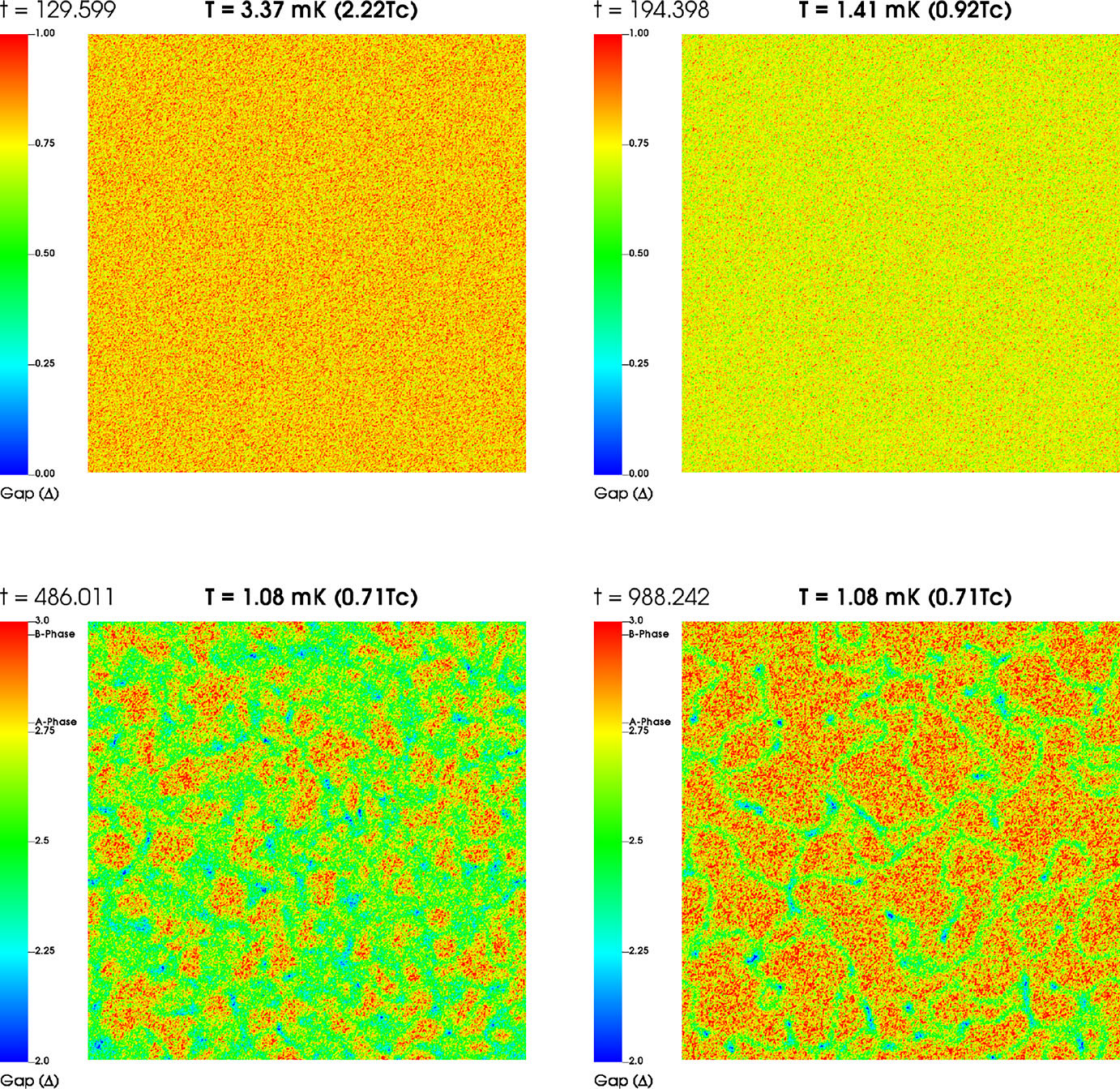
Snapshots of the order parameter gap during the quench. Top left: initial state at high temperature $T=3.37$ mk ($2.22\ T_\text {c}$), showing a homogeneous disordered configuration. Top right: system near the end of the quench at $T=1.41$ mK ($0.92\ T_\text {c}$). Bottom left: onset of B-phase nucleation at $T=1.08$ mK ($0.71\ T_\text {c}$), where distinct domains begin to form. Bottom right: late-time configuration at the same temperature, displaying coarsened B-phase regions separated by B–B domain walls and the presence of vortices formed during the cooling process. In this panel, vortices are identified as localized regions of reduced gap magnitude (bluish features), while B–B domain walls are identified as the extended green line-like structures separating regions of different B-phase orientation

Results from the simulation performed are shown in Fig. 3, where the gap $\Delta = \sqrt{\text {tr} A^\dagger A}$ is shown for a slice of the simulation box. The gap is given in units of $\ k_\text {B}T_\text {c}$ and at the final temperature, $T_f$, the gap for the A-phase is $\Delta _A= 2.766\ k_\text {B}T_\text {c}$ and for the B-phase $\Delta _\textrm{B}= 2.966 \ k_\text {B}T_\text {c}$. We present four snapshots of the system: the initial state of the quench, the final stages of the quench, the onset of B-phase emergence, and a late-time configuration showing the B–B domain walls and vortices formed during the cooling process.

At the beginning of the quench, the system is initialized at a uniform temperature $T_i$ above the critical temperature $T_c$, such that the order parameter is close to zero throughout the simulation volume. However, the first snapshot shown in Fig. 3 corresponds to a later stage of the evolution at T=3.37 mK (2.22 $T_c$), where the system still remains in the normal phase. At this stage, the gap is essentially absent apart from small-amplitude fluctuations, and no explicit phase bias or thermal inhomogeneity is imposed.

As the quench proceeds, the system initially remains approximately homogeneous, reflecting the finite response time of the order parameter to the rapid cooling. The second snapshot in Fig. 3, taken at T=1.41 mK (0.92 $T_c$), shows that the gap has only weakly developed and remains largely uniform across the system, indicating that the system is still in the early growth regime.

At later times, the order parameter increases significantly and develops pronounced spatial structure. The third snapshot, at *T* = 1.08 mK (0.71 $T_\textrm{c}$), shows the onset of B-phase nucleation, with distinct domains emerging from the initially homogeneous background. In the final snapshot, taken at a later time at the same temperature, these domains have coarsened into extended B-phase regions separated by B–B domain walls, and vortices are clearly visible as topological defects generated during the cooling process.

These homogeneous quench simulations provide an important reference point for interpreting more complex scenarios involving localized heating, composed initial conditions, and non-trivial boundary conditions. In particular, they allow us to disentangle genuinely non-equilibrium bulk dynamics from effects induced by thermal gradients or confinement. In future work, we will build on this baseline by introducing spatially inhomogeneous temperature profiles and boundary effects, which will lead to richer dynamics and more direct connections to experimental realizations of the A–B transition.

## Summary and Outlook

In this work, we have presented dyGiLa, a massively parallel numerical framework for simulating non-equilibrium phase transition dynamics in superfluid $^3$He based on a time-dependent Ginzburg–Landau (TDGL) description. Motivated by the long-standing discrepancy between homogeneous nucleation theory and experimental observations of the A–B transition, dyGiLa adopts computational strategies originally developed for cosmological phase transitions to address the inherently dynamical, out-of-equilibrium nature of the problem. By enabling large-scale real-time simulations of the superfluid order parameter on three-dimensional lattices, the code provides a powerful tool to investigate defect formation, phase coexistence, and interface dynamics beyond equilibrium assumptions.

A central aspect of the present implementation is the self-consistent evolution of both the order parameter and the temperature field, reflecting the explicit temperature dependence of the TDGL coefficients. This feature allows dyGiLa to naturally model a wide range of experimentally relevant scenarios, including rapid quenches, localized heating events, and controlled cooling protocols. In addition, the flexible construction of initial and boundary conditions–applied independently to each face of the simulation domain–makes it possible to mimic confined geometries, phase-selective surfaces, and asymmetric thermal environments. Together, these capabilities significantly extend earlier numerical approaches and provide a systematic framework for exploring how non-uniform temperature profiles and boundary effects influence the stability and decay of the metastable A phase.

The simulations presented in this work demonstrate the emergence of rich non-equilibrium behavior following rapid quenches from the normal phase into the B phase, including the formation of spatially inhomogeneous structures and complex phase boundaries. In particular, we observe the growth of B phase regions and the formation of B-B domain walls and vortices [39] consistent with the Kibble–Zurek mechanism during the cooling process. The complexity of the wall-vortex system reflects the high dimension of the *p*-wave order parameter, and the emergence of isolated vortices in the superheated region created by the reaction of a $^3$He atom with a neutron [16, 17] seems to be a much more complex phenomenon than in simulations of the single complex field of an *s*-wave order parameter [59, 60]. The extra complexity may shed light on the discrepancy between the *s*-wave simulations and the experiments. The complex wall-vortex structures can also be expected in rapidly cooling B-phase regions following energy injection into the metastable A phase, and hence have relevance for the A–B transition.

The results further reinforce the close analogy between superfluid $^3$He and cosmological first-order phase transitions, where defect-mediated dynamics and out-of-equilibrium field evolution play a crucial role. In this sense, superfluid $^3$He continues to serve as a unique laboratory for exploring phenomena that are otherwise difficult to probe in the early Universe.

Looking ahead, dyGiLa opens several promising directions for future research. Ongoing and planned work within the QUEST-DMC collaboration aims to directly confront simulation results with experimental data, with the goal of identifying the dominant nucleation pathways under different pressure, temperature, and boundary conditions. Extensions of the framework to include more realistic container geometries, refined models of dissipation and noise, and longer dynamical evolutions will further enhance its predictive power. In parallel, systematic parameter scans enabled by large-scale HPC resources will allow the exploration of rare events and statistically robust transition rates.

More broadly, the transfer of numerical techniques between cosmology and condensed-matter physics exemplified by dyGiLa highlights the value of cross-disciplinary approaches to complex non-equilibrium phenomena. Beyond addressing the A–B transition puzzle in superfluid $^3$He, the methods developed here may inform theoretical modeling of first-order phase transitions in the early Universe, including those relevant for gravitational-wave production and dark-matter scenarios. In this way, dyGiLa provides not only a new tool for superfluid physics, but also a bridge between laboratory experiments and fundamental questions in cosmology.

## Data Availability

The code used to generate the data is publicly accessible at https://dygila.github.io
